# Molecular Detection and Antibiogram Profiling of *Pasteurella multocida* Isolated From Breeder Chickens Suspected of Fowl Cholera in Gondar City, Ethiopia

**DOI:** 10.1155/ijm/8356389

**Published:** 2025-04-21

**Authors:** Abdo Megra Geda, Aregash Wendimu, Solomon Lulie, Bereket Dessalegn, Liyuwork Tesfaw, Eyob Assefa, Kenaw Birhanu, Getaw Deresse, Dawit Dufera, Gashaw Enbiyale, Mulusew Tesfaye, Tadesse Mihret

**Affiliations:** ^1^Department of Veterinary Pathobiology, College of Veterinary Medicine and Animal Sciences, University of Gondar, Gondar, Ethiopia; ^2^Department of Veterinary Clinical Medicine, College of Veterinary Medicine and Animal Sciences, University of Gondar, Gondar, Ethiopia; ^3^Department of Veterinary Bacteriology and Molecular Biology, National Veterinary Institute, Bishoftu, Ethiopia

**Keywords:** antibiogram, breeder chickens, *capA*, fowl cholera, Gondar City, *hyaD/hyaC*, isolation, *Pasteurella multocida*

## Abstract

Fowl cholera is a highly infectious bacterial disease in poultry production. It is caused by *Pasteurella multocida* (*P. multocida*) and leads to significant health risks and financial losses. Therefore, this study is aimed at isolating, molecularly detecting, and analyzing the antibiogram of *P. multocida* from breeder chickens in Gondar City. A cross-sectional study design with purposive sampling was employed to collect a total of 130 tracheal swab samples from breeder chickens showing clinical signs of fowl cholera between January 2023 and December 2023, based on case availability. Bacterial isolation was performed using bacteriological and biochemical tests. The isolated *P. multocida* was confirmed through conventional polymerase chain reaction (PCR) using a capsular serotype-specific primer (*capA*). The antibiogram assessment of *P. multocida* against 10 antimicrobial agents was conducted using the Kirby–Bauer disk diffusion method. Descriptive statistics were used to analyze the isolation rate of the bacterium. Of the 130 sampled swabs, 10 (7.69%) tested positive for *P. multocida* in the phenotypic assay, and 3 (30%) of those isolates were positive for the *hyaD/hyaC* virulence gene. The study found that all three isolates were 100% sensitive to penicillin, ampicillin, norfloxacin, and florfenicol, while showing 100% intermediate sensitivity to streptomycin and 66.7% intermediate sensitivity to gentamycin, amoxicillin, tetracycline, trimethoprim/sulphamethoxazole, and kanamycin. The study confirms that *P. multocida*, the causative agent of fowl cholera in breeder chickens, is circulating in the area and exhibits varying antimicrobial sensitivity profiles.

## 1. Introduction

Ethiopia has an estimated chicken population of 57 million, consisting of 78.84% indigenous breeds, 12.05% hybrids, and 9.11% exotic breeds [1]. This diverse chicken population plays a significant role in the country's economy [2]. Chicken plays a crucial role in providing high-quality animal protein, addressing malnutrition, improving nutritional status, and supplying meat and eggs for consumption [3]. They also serve as a vital source of cash income for nearly 80% of the Ethiopian population through the sales of live chicken and eggs [4, 5].

However, chicken production in Ethiopia faces significant challenges, primarily due to diseases such as fowl cholera, caused by the highly contagious Gram-negative bacterium *Pasteurella multocida* (*P. multocida*) [6, 7]. Fowl cholera is an endemic and often affects the respiratory tracts of chickens, turkeys, ducks, and geese [8, 9]. The disease results in economic losses due to death, weight loss, carcass condemnation, and increased medication costs for chicken producers [10]. It is estimated that fowl cholera causes 40%–60% mortality in newly hatched chicks, with morbidity and mortality rates reaching 52% and 56%, respectively [9].

*P. multocida* typically resides as a commensal organism in the respiratory tracts of various avian species but can cause fowl cholera when chickens are exposed to stress, poor health, or unfavorable environmental conditions [11]. The severity of fowl cholera is linked to virulence genes, particularly *hexA* and *hyaD/hyaC* genes. The *hexA* gene is involved in the capsule production, while *hyaD/hyaC* contributed to the lipopolysaccharide outer core, enhancing the bacterium's virulence and enabling it to resist the chicken's immune response and promote infection [12–14]. The *hyaD/hyaC* gene also facilitates the heme uptake system, allowing *P. multocida* to use heme as an iron source, scavenge iron from the host's tissues, and enhance its virulence and ability to establish infection in the chicken [13, 15].

Fowl cholera can present in acute or chronic forms, displaying various clinical symptoms. These may include swelling and darkening of the wattles and comb, facial edema, oronasal and ocular discharges, localized inflammation, dyspnea, sudden death without prior symptoms, lack of coordination, and grayish diarrhea depending on the form of the disease [16, 17].

The symptoms and lesions in fowl cholera in chickens resemble those of other bacterial diseases, such as Infectious coryza, colibacillosis, and salmonellosis [18]. Thus, its diagnosis requires identifying specific clinical signs and performing a microscopic examination to observe bacteria with bipolar staining in tissue smears from the liver, lung, and heart. Bacteriological tests, including assessments of cultural morphology, Gram staining, and biochemical characteristics of *P. multocida*, are essential following clinical evaluation [19]. Molecular techniques, such as polymerase chain reaction (PCR) assays, capsular and lipopolysaccharide genotyping, and virulence genotyping, are commonly used for differentiating *P. multocida* due to their application of specific primers. Deoxyribose nucleic acid (DNA) sequencing is also highly recommended for detecting *P. multocida* in chickens infected with fowl cholera [20, 21].

Although antibiotics are commonly used to treat fowl cholera, their overuse has led to *P. multocida* developing resistance to many antimicrobials [22]. This highlights the importance of regularly screening for antibiotic sensitivity and adjusting treatment as necessary.

Research studies in Ethiopia have reported the presence and prevalence of fowl cholera in chickens across various regions. For instance, Molalign reported an 80% prevalence of fowl cholera during a disease outbreak [23], and Chaka documented a 65% prevalence of *P. multocida* in apparently healthy chickens at markets in various districts of the Eastern Shewa Zone [24]. Additionally, Asfaw found a 27.5% prevalence of fowl cholera across different Ethiopian regions from 2018 to 2019 [10].

Despite fowl cholera being considered as one of the most prevalent diseases [25], there is a significant lack of information regarding its circulation in Gondar City. In this area, antibiotics are often used based on tentative clinical diagnosis, resulting in misuse and ineffective treatment of the disease. Furthermore, there has been limited research on molecular detection and antibiogram patterns of *P. multocida* causing fowl cholera in chickens in the city. These knowledge gaps hinder effective disease control and prevention strategies, negatively impacting chicken producers, consumers, market value, and the overall socioeconomic situation. As a result, reliable and timely diagnosis based on clinical findings, cultural isolation, molecular confirmation, and phenotypic assessment of antimicrobial resistance in the study area is essential. The findings of this study provide valuable information for veterinarians working in clinics, helping them understand fowl cholera, its clinical aspects, and laboratory analyses in chickens. Assessing the antibiogram of *P. multocida* allows veterinarians to choose the most effective antibiotics for treating fowl cholera, which can reduce chicken mortality and improve treatment strategies. This research also provides a foundation for future studies on the topic. Therefore, the aim of this study was to isolate *P. multocida*, conduct molecular detection, and evaluate its antibiogram in breeder chickens suspected of having fowl cholera in Gondar City.

## 2. Materials and Methods

### 2.1. Study Area and Population

**Figure 1:**
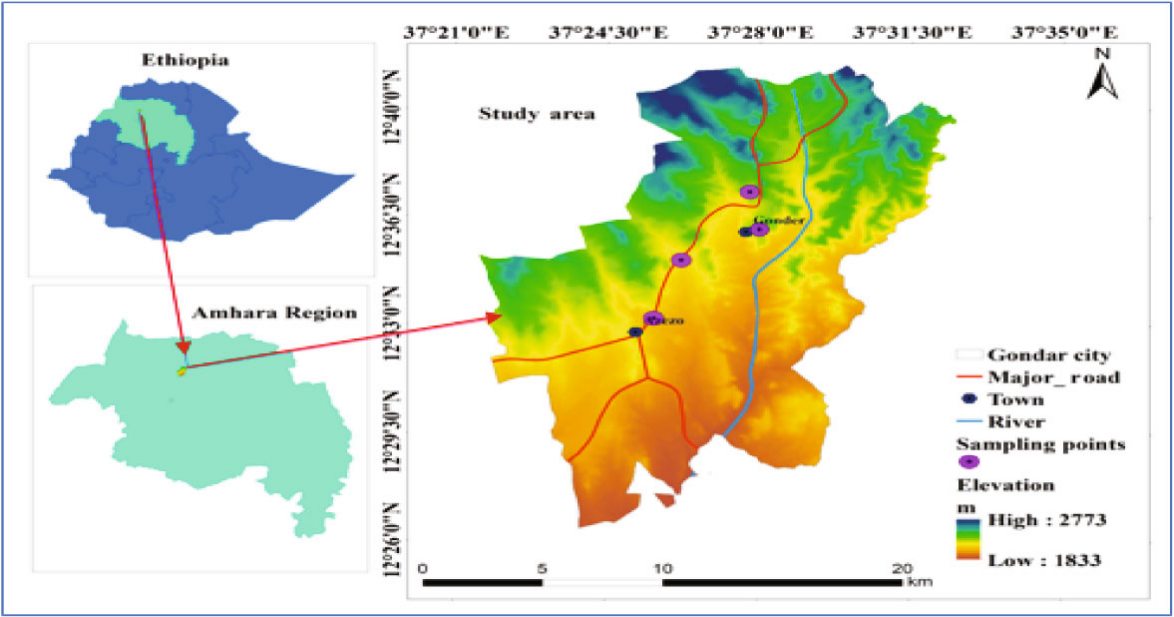
Map of the study site, Gondar City, created by ArcGIS.

The study was conducted in Gondar City, which is the capital city of the central Gondar Zone of Amhara regional state, Ethiopia (Figure 1). Gondar City is located in the Northwest part of Addis Ababa at a distance of 740 km. The city is situated at approximately 37°21⁣′–37°35⁣′ E longitude and 12°26⁣′–12°40⁣′ N latitude. Its elevation ranges from 1833 to 2773 m. Moreover, the city experiences an average annual temperature of 19.3°C and a mean annual rainfall of 1000 mm. Additionally, the city's relative humidity ranges from 30% to 40% in the dry season and from 60% to 70% in the rainy season [26].

There are approximately 19.1 million chickens in the Amhara region, of which 3.2 million are found in the central Gondar zone. Similarly, about 816,872 chickens are located in the city, and this chicken population supports an estimated 487,224 residents of Gondar City in their livelihoods [1]. Furthermore, the city has significant potential for chicken production, agricultural activities, and crop cultivation [7].

The study population consisted of chickens exhibiting clinical signs of fowl cholera, including coughing, sneezing, dyspnea, nasal discharge, tracheal rales, yellowish-gray diarrhea, swelling and blackening of comb, and wattles [27]. Hence, the chickens were selected for sampling without considering their age, sex, and breed. All chickens were handled humanely and received standard veterinary care during their visits.

### 2.2. Study Design and Sample Size

This cross-sectional study used a purposive sampling technique to collect clinical samples between January 2023 to December 2023. Samples were collected from regions with breeder chicken populations experiencing repeated occurrences of the disease in and around Gondar City. Thus, a total of 130 chickens showing clinical signs of fowl cholera were collected from each sampling point of the study area based on the availability of cases: University of Gondar (UoG) Veterinary Teaching Hospital (*n* = 59), Azezo Market Center (*n* = 23), Hewariaphawulos Veterinary Clinic (*n* = 37), and Arada Market Center (*n* = 11).

### 2.3. Sample Collection and Transportation

Samples from breeder chickens suspected of having fowl cholera were aseptically collected by taking tracheal swabs from each diseased chicken using sterile, moistened cotton swabs [28]. The swabs were placed in sterile universal test tubes containing 3 mL of tryptone soya broth (Himedia, India) as a transport medium. The collected samples were properly labeled and transported in an icebox to the veterinary microbiology laboratory at the UoG, College of Veterinary Medicine and Animal Sciences for microbiological analysis. Upon arrival, the samples were processed immediately and stored at + 4°C until further processing was required [29].

### 2.4. Bacteriological Isolation of *P. multocida*

For isolation of *P. multocida*, the collected swab sample was streaked aseptically on blood agar (Himedia, India) supplemented with 5% defibrinated sheep blood following bacteriological standard techniques [30]. The inoculated plate was incubated at 37°C for 24 to 48 h to check the hemolytic characteristic of *P. multocida*.

Subsequently, selective subculture was performed again on blood agar to obtain a pure culture [31]. Based on the colony characteristics (tiny, nonhemolytic with a shiny appearance resembling mucus on a dewdrop), a single isolated colony was picked and Gram-stained to confirm the bacterial morphology [32].

All cultures with Gram-negative appearance, small, smooth, or rough, with bipolar cocco-bacilli characteristics were subcultured again on blood agar (Himedia, India) to assist in bacterial identification for further analysis. Thus, the same colony was cultured on blood agar, again on MacConkey agar (Himedia, India) plates, and incubated at 37°C for 24–48 h. *P. multocida* isolates were selected based on the cultural characteristics of the blood agar and MacConkey agar. Isolates that grew on blood agar (Himedia, India), without hemolysis, and did not grow on MacConkey agar (Himedia, India) were considered *P. multocida*. For further analysis, the isolates were then subcultured into Tryptone soya agar (Himedia, India) from blood agar and stored at −20°C for delayed analysis.

### 2.5. Biochemical Characterization of *P. multocida*

*P. multocida* isolates were characterized by various biochemical tests, including catalase, oxidase, indole (Himedia, India), citrate (Himedia, India), and urease (Accumix, India), to assess the specific enzymatic reaction. Additionally, triple sugar iron (Himedia, India) was used to evaluate the metabolic capabilities of the bacterium. To further assess the ability of *P. multocida* to metabolize various sugars, different sugar fermentation tests were conducted. These sugars included lactose (Carelabmed, India), sucrose (Loba Chemie Pvt. Ltd, India), mannitol (Blulux Laboratories Pvt., Ltd India), dulicitol (HiMedia laboratories Pvt., Ltd, India), sorbitol (Loba Chemie Pvt. Ltd, India), glucose (Loba Chemie (P) Ltd, India), galactose (UMI-CHEM, India), arabinose (Finkem laboratories Pvt., Ltd, South Africa), and maltose and mannose (Blulux Laboratories (P) Ltd, India). For the sugar fermentation tests, 1% of each sugar was used in a buffered peptone water medium (Alpha Chemika, India), incorporating it with 1% of phenol red as an indicator [19, 33].

To maintain a long-term stock of *P. multocida,* the bacteria were inoculated onto slanted BHI agar (Himedia, India) and incubated aerobically at 37°C overnight. To improve preservation and facilitate transportation, a sterile 98% glycerol overlay was applied to the slanted agar.

### 2.6. Molecular Detection of *P. multocida* Virulence Gene

#### 2.6.1. Extraction of DNA

DNA extraction from *P. multocida* isolates was carried out at the molecular biology laboratory of the National Veterinary Institute (NVI), Bishoftu, Ethiopia. Briefly, 10 isolates exhibiting presumptive characteristics of *P. multocida* were selected and cultured aerobically in tryptone soya broth (Himedia, India) for 24–48 h at 37°C. The DNA was extracted using the snap chill technique. A 1500-*μ*L volume of cultured broth was combined with 200 *μ*L of deionized water in 1.8 mL Eppendorf tubes [34]. The bacterial cells were lysed by boiling for 10 min, followed by immediate cooling on ice for 10 min. The nucleic acid was separated by centrifuging the lysate at 13,000 rpm for 10 min [35]. The supernatant containing the DNA was collected and kept at −20°C until further use.

The purity of the extracted DNA was assessed using a NanoDrop (Thermo Fisher Scientific) to ensure sample quality. A ratio of 1.7–1.9 between absorbance readings at 260 and 280 nm is indicative of pure DNA [36].

#### 2.6.2. Detection of Virulence Gene by Conventional PCR

The PCR protocol was applied to isolates presumptively identified as *P. multocida* for PCR analysis [37]. The PCR assays were conducted to amplify the bacterial fragment using specific primers targeting the capsular biosynthesis gene (*hyaD/hyaC*) of *P. multocida* (Table 1) [38]. As outlined in Table 2, this process followed the PCR cycle and incorporated the necessary components of the PCR reaction. During the assay, the standard reference strain of *P. multocida* Serogroup A from the NVI was also used as a positive control, while the negative control contained no template DNA. The PCR products were then analyzed by agarose gel electrophoresis to visualize the amplified fragments.

#### 2.6.3. Visualization of PCR Products Through Agarose Gel Electrophoresis

To visualize the amplified PCR products, agarose gel electrophoresis was performed using a 2% w/v agarose gel in 0.5X Tris-borate ethylene diamine tetra acetic acid (EDTA) buffer. The gel was stained with 0.5 *μ*g/mL ethidium bromide for 10 min to facilitate the visualization of the DNA bands [39]. Each PCR product, combined with 6× loading dye, was loaded onto separate wells of the premade gel in a 5 *μ*L volume. To estimate the size of the amplified fragments, 1 kb plus DNA molecular markers was loaded in the first and last lanes. The gel was run at 120 V for 60 min on an electrophoresis machine (EC 2060, United States). Afterward, the gel was examined under ultraviolet light using a gel documentation system (Alpha imager, Germany), and the resulting bands were photographed for further analysis.

### 2.7. Antimicrobial Susceptibility of *P. multocida*

The in vitro antimicrobial susceptibility of *P. multocida* isolates was determined using the Kirby–Bauer disk diffusion method on Mueller–Hinton agar (Himedia, India), following Clinical and Laboratory Standard Institute (CLSI) guidelines [40]. Based on the standard guidelines and effectiveness in treating fowl cholera in chickens, 10 antimicrobial agents (Condalab, Spain) were selected: amoxicillin (AML) (30 *μ*g), penicillin (P) (10 *μ*g), gentamycin (CN) (10 *μ*g), ampicillin (AMP) (10 *μ*g), trimethoprim/sulphamethoxazole (SXT) (25 *μ*g), norfloxacin (NOR) (10 *μ*g), tetracycline (TE) (30 *μ*g), kanamycin (K) (30 *μ*g), streptomycin (S) (10 *μ*g), and florfenicol (FFC) (30 *μ*g) (Table 3).

During the test, approximately 4–5 similar colonies were cultured in tryptone soya broth (Himedia, India) supplemented with 10% horse serum to promote bacterial growth. The cultures were incubated for 2–8 h to obtain a pure bacterial culture. This suspension was then used to resuspend the bacteria in saline, adjusting turbidity to match 0.5 McFarland standards. After adjustment, the *P. multocida* suspension was evenly spread over the surface of a Mueller–Hinton agar plate using a sterile cotton swab. The plates were left upside down to dry for a few minutes before antibiotic disks were placed on them using sterile forceps. Each antibiotic disk was spaced 3 cm apart and 1.5 cm from the plate's edge. The plates were then incubated at 37°C for 8–12 h. After incubation, the diameters of the inhibition zone were measured to the nearest millimeter using an automatic caliper. The results of the isolates were then interpreted and classified as resistant, intermediate, or susceptible according to the CLSI guidelines [40].

### 2.8. Data Management and Analysis

The collected data were first coded and entered into a Microsoft Excel 2010 spreadsheet by the research team. Descriptive statistics, including frequency and percentage, were used to summarize the isolation rate of *P. multocida* based on the biochemical and PCR test results following primary cultural characterization.

## 3. Results

### 3.1. Isolation and Identification of *P. multocida*

**Figure 2:**
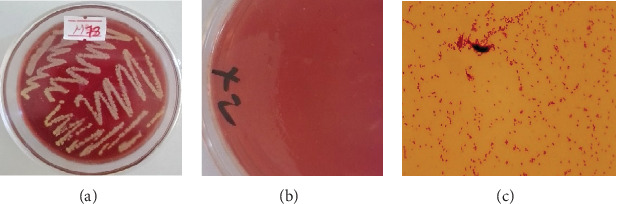
Growth patterns and Gram staining properties of *P. multocida*. (a) The growth of *P. multocida* on blood agar. (b) MacConkey agar did not support bacterial growth. (c) The Gram staining result for *P. multocida*.

In this study, the overall isolation rate of *P. multocida* from tracheal swabs of fowl cholera-suspected chickens, based on cultural characteristics, was 7.69% (10/130). The bacteria formed nonhemolytic, mucoid, convex, whitish-gray, shiny colonies resembling dew drops on blood agar (Figure 2a). However, the bacterium did not grow on MacConkey agar, as the medium does not support its growth (Figure 2b), which is a characteristic of *P. multocida*. Additionally, Gram stain revealed a Gram-negative appearance, with small, cocco-bacilli appearing singly, in pairs, or in short chains, further suggesting *P. multocida* (Figure 2c).

**Figure 3:**
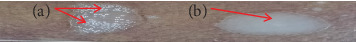
Reaction of *P. multocida* in the catalase test: (a) positive reaction for *P. multocida*; (b) the negative control.

**Figure 4:**
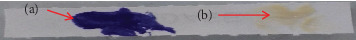
Reaction of *P. multocida* in the oxidase test: (a) a positive reaction for *P. multocida*; (b) a negative culture.

**Figure 5:**
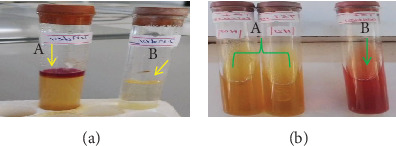
Reaction of *P. multocida* in the (a) indole test and (b) TSI test, respectively. The indole test shows (A in panel (a)) a positive reaction for *P. multocida* and (B in panel (a)) a negative culture. In the TSI agar test, *P. multocida* also demonstrates (A in panel (b)) a positive reaction alongside (B in panel (b)) a negative culture.

**Figure 6:**
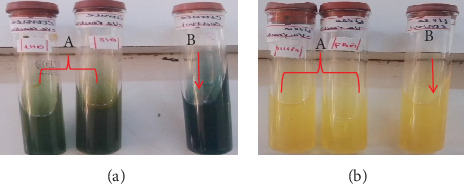
Reaction of *P. multocida* on (a) Simmons citrate and (b) urea test. *P. multocida* showed a negative reaction for the citrate test ((A in panel (a)) both test tubes), which is consistent with the uninoculated culture used as (B in panel (a)) a negative control. Similarly, in the urease test, *P. multocida* also demonstrated a negative reaction ((A in panel (b)) both test tubes), consistent with (B in panel (b)) the uninoculated tube.

**Figure 7:**
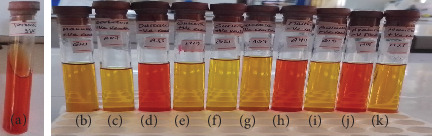
Reaction of *P. multocida* in 10 sugar fermentation tests, including (a) a negative control. After incubation, *P. multocida* exhibited a positive reaction for (b) mannose, (c) sorbitol, (e) glucose, (f) sucrose, (g) lactose, (i) galactose, and (k) mannitol, while showing nonfermentation for (d) dulicitole, (h) maltose, and (j) arabinose.

In biochemical assays, the isolates of *P. multocida* exhibited positive reactions for catalase, as indicated by the production of effervescence in Figure 3, but not in the negative control (Figure 3). The oxidase test also gave a positive result, with a deep blue color in Figure 4, and none in the negative control (Figure 4). The isolates tested positive for indole, forming a red ring at the top of the culture in Figure 5 (A) but not in the negative control (Figure 5). They also showed a positive reaction in the triple sugar iron test, excluding H_2_S and gas production, as seen on the right side of Figure 5 and the left test tube, but not in the negative control (Figure 5). However, the isolates of *P. multocida* did not react with the citrate (Figure 6, A) or urease tests (Figure 6, B), with the controls shown in Figure 6, respectively. Additionally, based on their ability to ferment mannitol, mannose, sucrose, sorbitol, glucose, galactose, and lactose, as well as their inability to ferment maltose, arabinose, and dulicitol, all isolates were identified as *P. multocida* (Figure 7).

Relatively, the isolation rate of *P. multocida* showed high variation across different sampling points of the study area. For example, a high isolation rate of 10.17 was observed at the UoG Veterinary Teaching Hospital, followed by 8.11% at the Hawariaphawulos Veterinary Clinic (Table 4).

### 3.2. Molecular Detection of *P. multocida* Virulence Gene

**Figure 8:**
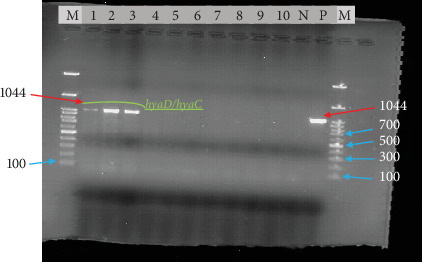
PCR result of *P. multocida* using a capsular serotype-specific primer (*cap*A). Note: Lanes 1, 2, and 3 showed a distinct band around 1044 bp, indicating positive results for *P. multocida*, while Lanes 4, 5, 6, 7, 8, 9, and 10 were negative. M = DNA molecular markers started at 100-bp; N = negative control; P = reference positive control of *P. multocida*.

Out of the 10 phenotypically identified *P. multocida* isolates, 30% (*n* = 3), or 2.31% (3/130) of the total samples, were positive for the *P. multocida* virulence genes (*hyaD/hyaC*) (Figure 8). All of these isolates were exclusively from the UoG Veterinary Teaching Hospital compared to other sampling points in the study area, and this variation might be associated with the number of chickens sampled.

### 3.3. Antibiogram Profile of *P. multocida*

**Figure 9:**
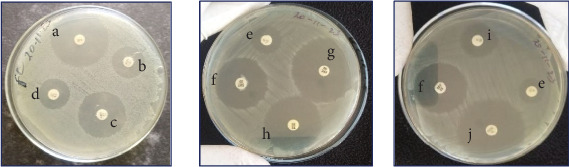
Antibiogram result of *P. multocida* isolates. Note: a = penicillin (10 *μ*g); b = amoxicillin (30 *μ*g); c = ampicillin (10 *μ*g); d = gentamycin (10 *μ*g); e = streptomycin (10 *μ*g); f = trimethoprim/sulphamethoxazole (25 *μ*g); g = florfenicol (30 *μ*g); h = tetracyclin (30 *μ*g); i = kanamycin (30 *μ*g); and j = norfloxacin (10 *μ*g).

All the tested isolates of *P. multocida* in this study (*n* = 3) showed 100% susceptibility to P (10 *μ*g), AMP (10 *μ*g), FFC (30 *μ*g), and NOR (10 *μ*g) while exhibiting 100% intermediate to S (10 *μ*g) (Figure 9). Furthermore, the results of the antibiogram patterns of *P. multocida* isolates were summarized in Table 5.

## 4. Discussion

This study was conducted to determine the presence of *P. multocida* causing fowl cholera in Gondar City, Ethiopia, using culture-based and molecular techniques. In this study, three *P. multocida* isolates were molecularly identified from 10 culturally characterized isolates out of 130 samples. These isolates were reported from the UoG Veterinary Teaching Hospital. This variation may be attributed to the low number of chickens sampled from Hawariaphawulos Veterinary Clinic, Azezo Market, and Arada Market Center, while the flow of cases was high in the UoG Veterinary Teaching Hospital.

The culture techniques in this study revealed an overall isolation rate of 7.69% from the tracheal swabs of chicken suspected of fowl cholera. This result is similar to a study conducted in Kenya, where *P. multocida* was isolated from healthy chickens and market slaughter slabs at a rate of 6.2% [41]. However, our findings differ from Abood et al.'s study, which reported a lower isolation rate of 2.6% from layers showing signs of fowl cholera in Egypt [37]. This variation could be due to factors such as the number of samples tested, the season of sample collection, and the method used for isolation and identification.

Through PCR assays, 30% (3/10) of the isolates were confirmed as *P. multocida*. This finding differs from a study conducted in Bangladesh, which reported an 11.42% (4/35) detection rate [28]. The discrepancy may be due to differences in the sampling sites or sample types. Our study focused on tracheal swabs, while the Bangladesh study used samples from the spleen, liver, lung, and heart for *P. multocida* isolation and detection.

On the other hand, this study focused on chicken cases suspected of fowl cholera, which exhibited diagnostic clinical signs in the study area. However, this finding contrasts with studies by Panna et al. [28], Laban et al. [31], and Mbuthia et al. [41], which investigated apparently healthy chicken flocks to assess the carrier status of *P. multocida*. In our study, trachea swabs were used to isolate *P. multocida*, a method that aligns with the finding of Mehmood et al. [42], who also isolated the bacterium from the same sample type. Similarly, studies by Paudel et al. [43], Paudel et al. [44], Sorour et al. [45], and Atere Ayowole Victor et al. [46] successfully isolated *P. multocida* from trachea swabs, and these findings are consistent with our results.

Regarding antimicrobial susceptibility, the *P multocida* isolates in this study showed 100% susceptibility to P (10 *μ*g), AMP (10 *μ*g), FFC (30 *μ*g), and NOR (10 *μ*g). They also exhibited 100% susceptibility and 66.7% intermediate susceptibility to S (10 *μ*g) and CN (10 *μ*g), respectively. This is in agreement with the finding of Qandoos et al. [47], who also reported intermediate susceptibility of *P. multocida* to S and CN.

In a similar way, two isolates were 66.7% intermediate to tetracyclin (30 *μ*g) and SXT (25 *μ*g), while the remaining isolate was 33.3% susceptible to CN (10 *μ*g), AML (30 *μ*g), and K (30 *μ*g). Despite the small sample size (*n* = 3) in this study, these results are relatively consistent with Abood et al.'s study, which reported 75% sensitivity to CN and 100% sensitivity to AML in eight isolates from Egypt [37]. Rabana et al. in Nigeria also reported similar findings, where *P. multocida* isolates were susceptible to CN and SXT [48], as observed in our study.

However, our findings differ from those of Sabsabi et al., who found *P. multocida* isolates resistant to P (14%), FFC (23%), TE (37%), CN (14%), AML (14%), and S (68%) [49]. Similarly, Abood et al.'s findings differ from ours, as they reported 87.5% resistance to FFC and S [37]. Our current results also differ from those of Li et al. [17] and Zhu et al. [50], who found *P. multocida* isolates resistant to FFC, TE, S, K, and SXT. Additionally, El Demerdash et al. reported that *P. multocida* isolates from chickens were resistant to AMP and SXT [51], which does not align with the findings of our current study. These discrepancies may be attributed to variations in antibiotic exposure, as well as the presence or absence of antimicrobial resistance genes in *P. multocida*, and differences in antibiotic usage practices for treating the disease.

## 5. Conclusion and Recommendations

Fowl cholera, caused by *P. multocida*, is a common disease that affects chickens' respiratory systems and results in significant financial losses in the poultry industry. In this study, *P. multocida* was isolated from 7.69% of breeder chickens suspected of having the disease. Molecular analysis revealed that 3 of the 10 isolates contained the *P. multocida* virulence genes (*hyaD/hyaC*), all from the UoG Veterinary Teaching Hospital. These isolates were 100% sensitive to P, AMP, FFC, and NOR, indicating effective treatment options for fowl cholera. They also exhibited varying intermediate patterns to other antibiotics, with 100% intermediate to S and 66.7% intermediate to CN, TE, SXT, AML, and K. This study is the first in Gondar City to confirm the presence of *P. multocida*, making an important step in improving prevention and control strategies both in the city and beyond. Based on these findings, the researchers recommend further investigations, including bacterial DNA sequencing to gain better understanding of its genetic composition and potential virulence factors, research on antibiotic policies, and studies to raise community awareness. Additionally, they suggest conducting seroprevalence studies and identifying risk factors associated with the disease, extending the coverage to wider areas of Gondar City and other regions of the country.

## Figures and Tables

**Table 1:** Target gene, primers, and amplicon size for detecting *P. multocida*.

**Target gene**	**Primers**	**Sequence (5**⁣′**-3**⁣′**)**	**Size/bp**
*hyaD/hyaC*	*cap*A-fwd	5-TGCCAAAATCGCAGTCAG-3	1044
	*cap*A-rev	5-TTGCCATCATTGTCAGTG-3	

**Table 2:** Components of the PCR reaction and thermocycler conditions.

PCR reaction	Components	Each volume in *μ*L	Total volume in *μ*L	
	DH_2_O	8.5	25	
	2x PCR master mix (Promega, United States)	12.5		
	Target DNA template	2		
	10 pmol forward primer	1		
	10 pmol reverse primer	1		

PCR cycle	Event	Temperature in °C	Time	Number of cycles
	Initial denaturation	95	5 min	1
	Denaturation	95	30 s	
	Annealing	55		35
	Extension	72		
	Final extension	72	5 min	1

**Table 3:** Standard guidelines for antibiotic inhibition zone breakpoints for *P. multocida.*

**Antimicrobial agent**	**Disk potency in *μ*g**	**Diameter of the inhibition zone in mm**		
		**Susceptible**	**Intermediate**	**Resistant**
Amoxicillin	30	≥ 18	14–17	≤ 13
Penicillin	10	≥ 29	22–28	≤ 21
Gentamycin	10	≥ 15	13–14	≤ 12
Ampicillin	10	≥ 17	14–16	≤ 13
Trimethoprim/sulphamethoxazole	25	≥ 16	11–15	≤ 10
Norfloxacin	10	≥ 17	13–16	≤ 12
Tetracycline	30	≥ 19	15–18	≤ 14
Kanamycin	30	≥ 18	14–17	≤ 13
Streptomycin	10	≥ 15	12–14	≤ 11
Florfenicol	30	≥ 22	19–21	≤ 18

*Note: Source:* [40]. *μ*g, microgram; mm, millimeter.

**Table 4:** Presumptive isolation rates of *P. multocida* by sampling point in the study area.

**Sampling points**	**No. of the chickens examined**	**Positive (%)**
UoG Vet Teaching Hospital	59	6 (10.17)
Azezo Market Center	23	1 (4.35)
Hawariaphawulos Vet Clinic	37	3 (8.11)
Arada Market Center	11	0 (0.00)
**Total**	**130**	**10 (7.69)**

*Note:* %, percent; No., number; Vet, veterinary.

Abbreviation: UoG, University of Gondar.

**Table 5:** Result of antibiogram of *P. multocida* using Kibry–Bauer disk diffusion test.

**Antimicrobial disk**	**Disk potency (*μ*g)**	**Number of isolate/s**	
		**Susceptible**	**Intermediate**
Penicillin	10	3	0
Amoxicillin	30	1	2
Ampicillin	10	3	0
Gentamycin	10	1	2
Streptomycin	10	0	3
Trimethoprim/sulphamethoxazole	25	1	2
Florfenicol	30	3	0
Tetracycline	30	1	2
Kanamycin	30	1	2
Norfloxacin	10	3	0

*Note:μ*g, microgram; mm, millimeter.

## Data Availability

The original contributions presented in the study are included in the article materials. Further inquiries can be directed to the corresponding author.
